# Inter-observer agreement in interpreting chest X-rays on children with acute lower respiratory tract infections and concurrent wheezing

**DOI:** 10.1590/S1516-31802007000300005

**Published:** 2007-05-03

**Authors:** Carlos Bada, Nilton Yhuri Carreazo, Juan Pablo Chalco, Luis Huicho

**Keywords:** Thoracic radiography, Pediatrics, Diagnosis, Pneumonia, Bronchiolitis, Radiografía torácica, Pediatría, Diagnóstico, Neumonía, Bronquiolitis

## Abstract

**CONTEXT AND OBJECTIVE:**

Many children with acute lower respiratory tract infections (ALRI) present to the emergency ward with concurrent wheezing. A chest x-ray is often requested to rule out pneumonia. We assessed inter-observer agreement in interpreting x-rays on such children.

**DESIGNS AND SETTING:**

Prospective consecutive case study at Instituto de Salud del Niño, Lima, Peru.

**METHODS:**

Chest x-rays were obtained from eligible children younger than two years old with ALRI and concurrent wheezing who were seen in the emergency ward of a nationwide pediatric referral hospital. The x-rays were read independently by three different pediatric residents who were aware only that the children had a respiratory infection. All the children had received inhaled beta-adrenergic agonists before undergoing chest x-rays. Lobar and complicated pneumonia cases were excluded from the study.

**RESULTS:**

Two hundred x-rays were read. The overall kappa index was 0.2. The highest individual kappa values for specific x-ray findings ranged from 0.26 to 0.34 for rib horizontalization and from 0.14 to 0.31 for alveolar infiltrate. Inter-observer variation was intermediate for alveolar infiltrate (kappa 0.14 to 0.21) and for air bronchogram (kappa 0.13 to 0.23). Reinforcement of the bronchovascular network (kappa 0.10 to 0.16) and air trapping (kappa 0.05 to 0.20) had the lowest agreement.

**CONCLUSIONS:**

There was poor inter-observer agreement for chest x-ray interpretation on children with ALRI and concurrent wheezing seen at the emergency ward. This may preclude reliable diagnosing of pneumonia in settings where residents make management decisions regarding sick children. The effects of training on inter-observer variation need further studies.

## RESUMEN

**CONTEXTO Y OBJETIVO:**

Muchos niños con infecciones respiratorias agudas (IRA) bajas que se presentan a las unidades de emergencia tienen sibilancias concurrentes. En tales niños se solicita a menudo una radiografía de tórax para descartar neumonía. Realizamos un estudio para evaluar la variación inter-observador en la interpretación de las radiografías en niños con IRA baja y sibilancias concurrentes.

**TIPO DE ESTUDIO Y LUGAR:**

Estudio prospectivo de casos consecutivos realizado en el Instituto de Salud del Niño, Lima, Perú.

**METODOS:**

Se leyeron las radiografías de tórax de niños consecutivos elegibles menores de 2 años de edad con IRA baja y sibilancias concurrentes que acudieron a la emergencia de un hospital garbed de referencia en Lima, Perú. Las radiografías fueron leídas independientemente por 3 residentes de pediatría diferentes que habían recibido información clínica limitada sobre la presencia de una infección respiratoria. Todos los niños habían recibido agonistas beta-adrenérgicos inhalados antes de que se les tomara las radiografías de tórax. Las neumonías lobares y complicadas fueron excluídas del studio.

**RESULTADOS:**

Se leyeron 200 radiografías de tórax. El índice kappa global fue 0.2. Los valores kappa individuales más altos para hallazgos radiológicos específicos oscilaron entre 0.26 a 0.34 para horizontali zación de costillas y de 0.14 a 0.31 para infiltrado alveolar. La variación inter-observador fue intermedia para infiltrado alveolar (kappa 0.14 a 0.21) y para broncograma aéreo (kappa 0.13 a 0.23). l reforzamiento de la trama broncovascular (kappa 0.10 a 0.16) y el atrapamiento de aire (kappa 0.05 a 0.20) tuvieron la concordancia más baja.

**CONCLUSIONES:**

Hubo pobre concordancia inter-observador en la interpretación de las radiografías de tórax en niños con IRA baja y sibilancias concurrentes atendidos en la unidad de emergencia. Esto puede impedir un diagnóstico confiable de neumonía en lugares en los que los residents toman las decisiones de manejo de los niños enfermos. Se necesita realizar estudios adicionales para evaluar el efecto de la capacitación en la variación inter-observador

## INTRODUCTION

Acute lower respiratory tract infections (ALRI) in children are a leading cause of death and constitute a substantial burden of disease in developed and developing countries.^1,2^ A significant proportion of children with ALRI presenting to emergency wards may have also concurrent wheezing of varying severity.^3–6^

The World Health Organization (WHO) promotes a case-detection and antibiotic management policy for ALRI, particularly pneumonia.^7*^ Tachypnea and chest retraction are the key findings for making a diagnosis of pneumonia and putting patients on antibiotic therapy. There is strong evidence supporting the effectiveness of this policy in reducing childhood mortality due to pneumonia.^8,9^ However, in children with ALRI and wheezing, it is difficult to determine whether the difficulty in breathing is due to pneumonia or to bronchial obstruction underlying the wheezing. Physicians faced with these patients usually prescribe inhaled or nebulized beta-adrenergics and systemic corticosteroids, although the efficacy of these agents is questionable in infants and young children with bronchiolitis.^10,11^ In addition, if patients are not responsive to such treatment, an x-ray is often requested, and this is reportedly the ultimate criterion for defining the presence or absence of pneumonia. Physicians very often interpret x-rays as positive for pneumonia, although the majority of radiographs may not show a definite abnormality. This consequently leads to widespread use of antibiotics and increased frequency of hospitalization.

In an attempt to optimize management, the current ALRI case management guidelines recommend the administration of two cycles of rapid-acting bronchodilators at 15-minute interval to children with audible wheezing and fast breathing and/or lower chest indrawing. If there is improvement in the tachypnea and/or chest indrawing, they are sent home with oral bronchodilators.* The current generic guidelines for integrated management of childhood illness (IMCI) do not include management of children with wheeze,^12^ although country-level adaptations may have included this. Because little is known about the outcomes for these children, there are legitimate concerns regarding the efficiency of current ALRI case management guidelines for those children presenting ALRI with concurrent wheezing. These guidelines are based on audible wheezing (without medical auscultation), although it has been shown that audible wheezing is present in only 30% of children that have auscultatory wheezing.^13^ A recent study found that up to two thirds of children with wheeze are missed by these guidelines,^14^ and that as a consequence antibiotics are being overprescribed and bronchodilators underutilized. These authors concluded that children with wheeze constituted a special ALRI group requiring a separate management model.^14^ They further proposed that, in countries where wheezing is prevalent, it would be worthwhile training health workers in using stethoscopes to identify wheezing.

Unfortunately, there is not a universally agreed gold standard for discriminating between the presence of different types of pneumonia, particularly in those cases without defined alveolar infiltrate or lobar consolidation, which account for the majority of ALRI cases. A chest x-ray is the most commonly used criterion for defining pneumonia, but it has many drawbacks.^15^ These limitations include problems of the availability of radiographic facilities in most health services in the developing world, difficulty in interpreting chest radiographs from young children, wide variations in the experience and skill of x-ray interpreters, varying amounts and quality of clinical information available, inability of chest radiography to reliably distinguish viral from bacterial pneumonia, and inability of radiographs to reveal early changes in pneumonia.

Inter-observer agreement relating to chest x-rays in pediatric ALRI cases has been studied previously. A recent systematic review found 10 studies, but only six of them satisfied the inclusion criteria, and the quality of the methods and reporting was not consistently high.^16^ The authors concluded that there was little information on inter-observer agreement and there was insufficient information to assess the heterogeneity of agreement in different clinical settings. They highlighted the need for future studies that would pay attention to independent assessments of radiographs, include normal clinical populations of patients, describe the criteria for radiographic features and report on confidence intervals.

## OBJECTIVE

We are unaware of any clinical studies assessing the inter-observer agreement in interpreting chest x-rays from children with ALRI and concurrent wheezing presenting to emergency wards in settings where residents provide care for such children and make the diagnostic and therapeutic decisions. If the inter-observer agreement is poor, the usefulness of chest x-rays for diagnosing pneumonia may be seriously affected. Thus, the objective of this study was to assess the inter-observer variation in interpreting chest x-rays from preschool children with ALRI and concurrent wheezing who presented to the emergency ward of a pediatric hospital in Lima, Peru. The study focused on pediatric residents on duty at the emergency wards because children who are taken there receive initial care from these residents, who make most of the clinical decisions regarding diagnosis and management.

## METHODS

This study was performed at the emergency ward of Instituto Especializado de Salud del Niño in Lima, a national referral hospital that serves children from Lima and other cities in Peru.

Chest x-rays from children aged 1 to 23 months old with ALRI and concurrent wheezing that had lasted for less than 14 days were eligible. ALRI with concurrent bronchial obstruction was defined as a respiratory tract infection with coughing and/or difficult in breathing reported by the mother, and wheezing or ronchi or prolonged expiratory phase confirmed by physical examination.

Chest x-rays were excluded if they had lobar pneumonia or complicated pneumonia (empyema or parapneumonic pleural effusion). We also excluded chest x-rays from children with congenital heart disease (ventricular septal defect, atrial septal defect or persistent ductus arteriosus), cystic fibrosis, tracheobronchial congenital malformations (tracheomalacia, tracheobronchomalacia or bronchomalacia), tracheoesophageal fistula, vascular ring, Down's syndrome or immunodeficiency.

A pediatric resident on duty routinely assessed children with ALRI and concurrent wheezing who came to the emergency ward. Almost all these children received nebulized or inhaled beta-adrenergic agents as the initial therapy at the emergency ward. If the patients were not responsive to rescue therapy, systemic corticosteroid was administered and a chest-x ray was requested. The study design did not include interventions to modify this routine course of actions.

Chest x-rays from consecutive eligible children presenting to the emergency ward from November 2000 to May 2002 were included. Three different pediatric residents observed each x-ray independently. The observers had to complete a written questionnaire that included questions with closed answer alternatives relating to the descriptive characteristics of the x-rays, and the possible diagnoses included pneumonia, bronchiolitis, or both. Before each chest xray assessment, the observers were told that the child had an acute respiratory tract infection. No additional information was provided.

Each observer independently completed a form describing the x-ray, which included a statement about the presence or absence of any of the following: reinforcement of the bronchovascular network, air trapping, rib horizontalization, air bronchogram and alveolar infiltrate. We deliberately did not provide the residents doing the evaluations with any operational definition regarding the presence or absence of the specified characteristics. In this way, we attempted to reproduce an effectiveness result, i.e. the performance of residents in interpreting chest x-rays under routine working conditions, rather than assessing their efficacy under controlled conditions.

Additionally, the residents assigned each case to a diagnostic category, based on the x-ray findings and on the knowledge that the child had ALRI. The possible diagnoses included pneumonia, wheeze (bronchiolitis), and pneumonia plus wheeze.

All the children were followed up and the diagnosis made by each observer was compared with the final diagnosis. This final diagnosis was the one assigned to and recorded for each child after the observation period in the emergency ward or upon discharge for hospitalized children and, according to the clinical outcome, included the response to systemic corticosteroids and nebulized beta-adrenergic agonists. This information was gathered from the respective clinical records.

The inter-observer agreement rates between observers 1 and 2, observers 1 and 3, and observers 2 and 3 were determined by means of kappa index calculations for each of the x-ray characteristics evaluated. In addition, kappa indexes were calculated to assess the agreement rate between each observer's diagnosis and the discharge diagnosis, and the agreement between observers regarding their overall radiographic diagnoses. The Statistical Package for the Social Science (SPSS) software, version 11.0, was used for the analyses.

## RESULTS

Two hundred x-rays were independently assessed by three pediatric residents. Eighty-eight x-rays were from children who were ultimately hospitalized and 112 were from children who were ultimately discharged after a variable period of observation in the emergency ward.

The inter-observer agreement is shown in Table 1. The overall kappa index was 0.2. The highest individual kappa values for specific x-ray findings ranged between from 0.26 to 0.34 for rib horizontalization and from 0.14 to 0.31 for alveolar infiltrate. Inter-observer variation was intermediate for alveolar infiltrate (kappa values from 0.14 to 0.21) and for air broncho-gram (kappa from 0.13 to 0.23). The descriptive characteristics with the lowest agreement were reinforcement of the bronchovascular network (kappa from 0.10 to 0.16) and air trapping (kappa from 0.05 to 0.20). The kappa indexes for inter-observer agreement between observer diagnosis and final diagnosis ranged from 0.17 (95% confidence interval, CI: 0.075-0.278) to 0.27 (95% CI: 0.166-0.380). The kappa indexes for agreement between observers with respect to their overall radiographic diagnoses ranged from 0.17 (95% CI: 0.075-0.278) to 0.27 (95% CI: 0.166-0.380).

**Table 1.** Inter-observer agreement

	Bronchovascular network	Air trapping	Rib horizontalization	Alveolar infiltrate	Air bronchogram
Observer 1- Observer 2					
Kappa	0.101	0.13	0.292	0.215	0.239
p	0.151	0.063	**0.000**	**0.002**	**0.001**
95% CI	[-0.05, 0.26]	[-0.01,0.27]	[0.157, 0.426]	[0.081, 0.350]	[0.093, 0.385]
Observer 1- Observer 3					
Kappa	0.163	0.051	0.264	0.146	0.135
p	**0.021**	0.465	**0.037**	**0.037**	0.055
95% CI	**[0.004, 0.322]**	[-0.088, 0.191]	[0.128, 0.401]	[0.010, 0.282]	[**-0.008**, 0.278]
Observer 2- Observer 3					
Kappa	0.164	0.204	0.34	0.313	0.194
p	**0.02**	**0.004**	**0.000**	**0.000**	**0.006**
95% CI	**[0.001, 0.322]**	[0.066, 0.343]	[0.208, 0.472]	[0.181, 0.444]	[0.051, 0.338]

*CI = confidence interval.*

The descriptive findings and corresponding diagnoses are shown in Table 2. Air bronchograms were not used as a criterion for diagnosing pneumonia or bronchiolitis by any of the observers and thus they are absent from Table 2. Table 3 summarizes the frequencies of descriptive findings and diagnostic categories for each observer.

**Table 2.** Percentage of diagnostic categories against percentage of descriptive categories for each observer

	Bronchovascular network	Air trapping	RH	Alveolar infiltrate
		**Observer 1**		
Pneumonia				
Yes	16.07	8.95	11.2	29.90
No	33.33	38.80	31.57	6.38
Wheezing				
Yes	42.26	42.53	44.80	6.54
No	42.42	41.79	38.15	82.97
Both				
Yes	41.66	48.50	44	63.55
No	24.24	19.40	30.26	10.63
		**Observer 2**		
Pneumonia				
Yes	18.29	2.38	5.88	32.65
No	27.02	49.33	40.24	7.76
Wheezing				
Yes	48.78	58.73	52.10	7.14
No	51.35	33.33	45.12	89.32
Both				
Yes	32.92	38.88	42.01	60.20
No	21.62	17.33	14.63	9.70
		**Observer 3**		
Pneumonia				
Yes	21.47	7.69	10.15	36.08
No	31.57	52.11	46.57	11.53
Wheezing				
Yes	43.55	53.07	52.34	5.15
No	55.26	32.39	34.24	83.65
Both				
Yes	34.96	39.23	37.5	58.76
No	13.15	15.49	19.17	4.80

*RH = rib horizontalization.*

**Table 3.** Frequencies and proportions of diagnostic categories and x-ray descriptive findings, by observer. Numbers in parentheses are proportions

	Observer 1	Observer 2	Observer 3
**Diagnosis**			
Pneumonia	38 (19)	39 (19.5)	47 (23.5)
Wheezing	84 (42)	99 (49.5)	91 (45.5)
Pneumonia plus wheezing	78 (39)	62 (31)	62 (31)
**X-ray description**			
Bronchovascular network	168 (84)	163 (81.5)	163 (81.5)
Air trapping	134 (67)	126 (63)	129 (64.5)
Rib horizontalization	125 (62.5)	119 (59.5)	127 (63.5)
Alveolar infiltrate	107 (53.5)	97 (48.5)	97 (48.5)
Air bronchogram	56 (28)	52 (26)	62 (31)

## DISCUSSION

The overall inter-observer agreement and the inter-observer agreement for specific radiological findings were poor in this study. This is of concern, because the diagnostic usefulness of chest x-rays for children with ALRI is heavily dependent not only on the x-ray findings but also on the degree of concordance between observers. Moreover, in our emergency wards the pediatric residents are in charge of the initial diagnostic and therapeutic interventions and they make the decisions regarding discharging or admitting the patients. The definitive diagnosing of bacterial pneumonia is not an easy task, especially because of the low rate of isolation of etiological agents from blood cultures of children with pneumonia. Furthermore, the accuracy of serological tests for bacteria, chlamydiae and viruses is variable, and invasive procedures such as transthoracic lung puncture or transtracheal aspiration for culturing are unacceptable in daily practice.

A substantial proportion of children with ALRI presenting to our emergency wards have concurrent wheezing. Diagnosing with certainty whether a child with ALRI and wheeze also has pneumonia is more difficult if there is no clearly defined infiltrate or no consolidation in the chest-x ray. We did not include lobar pneumonia in our study because its diagnosis is easier and, in addition, the great majority of patients with breathing difficulty do not have pneumonia with a lobar consolidation. It is very difficult in this context to know whether bronchial obstruction or underlying pneumonia is responsible for the difficult breathing.^17^ Moreover, so far, no clinical clues or laboratory tests have been developed that are sufficiently accurate to reliably discriminate between whether the pneumonia is due to bacteria, viruses, chlamydiae or mycoplasma.^18–21^ Improvement of diagnostic aids for identifying the etiological agents in children with ALRI and concurrent wheeze could contribute towards improved antimicrobial use. In addition, it has been suggested that in settings where wheezing is common, training health workers in the use of stethoscopes for identifying wheezy children can improve ALRI case management.^14^ Furthermore, a recent report from Brazil demonstrated an association between persistent tachypnea after rescue administration of bronchodilators in children with a history of previous respiratory distress and presence of pulmonary infiltrate.^22^ These authors concluded that incorporation of a history of previous respiratory distress and response to bronchodilators may dramatically decrease the proportion of children receiving unnecessary antibiotics.

The criteria for making a diagnosis of pneumonia or bronchiolitis (wheeze) or both in the present study revealed inaccuracies in pediatric residents’ knowledge of the fundamentals of pediatric chest-x rays. The perceived presence of air trapping and hyperinflation was considered the basis for a diagnosis of bronchiolitis in 42% to 59% of the cases, whereas the presence of alveolar infiltrate and air bronchogram served as the justification for a pneumonia diagnosis in 23% to 68% of the cases. This highlights the need for intensive training for pediatric residents in the fundamentals of pediatric radiology, particularly in the aspects relating to acute respiratory infections. We acknowledge that it is difficult to determine with certainty whether the poor agreement found in our study is due to the inherent limitations of radiography or to inadequate training. The influence of the level of experience and of previous training on the reliability of chest x-ray interpretation has not being sufficiently studied, particularly in children with ALRI, and the results from published reports are not entirely consistent.^23–26^ We furthermore emphasize that physicians and particularly pediatric residents must take into account that chest-x rays constitute only one of the ways of reliably diagnosing pneumonia in children. Clinical assessments that encompass efficiently determining the symptoms and physical signs are of paramount importance for reaching a good diagnostic performance.^27^ Thus the need for good clinical training in pediatric residency must be underlined.

Additional important points relating to concurrence of pneumonia and wheezing that have previously been reported include a strong epidemiological association between pneumonia and asthma, bronchitis and other lung problems.^28,29^ Thus, the complexity of a clinical presentation that may include pneumonia and bronchial obstruction implies not only an underlying clinical challenge, but also interdependence between the two conditions.

## CONCLUSIONS

The inter-observer agreement for chest x-rays in young children with ALRI and concurrent wheezing was poor. In settings where residents are in charge of making clinical decisions, caution must be observed when using chest x-rays as the key diagnostic criterion for the presence or absence of pneumonia, and for decisions on discharge, admission or prescription of antibiotics in the emergency ward. The need for intensive training of pediatric residents in reading and interpreting chest x-rays from children with ALRI and concurrent wheezing must be emphasized, along with the need to further study the effect of a training period on the inter-observer rate. Nonetheless, the need for good clinical training should not be neglected.

## Footnotes

* WHO Programme for the Control of Acute Respiratory Infections. Technical basis for the WHO recommendations on the management of pneumonia at first-level health facility. Geneva: WHO; 1991 (unpublished document WHO/ARI/91.20).
